# Multilocus analysis of SNP and metabolic data within a given pathway

**DOI:** 10.1186/1471-2164-7-5

**Published:** 2006-01-13

**Authors:** Vessela N Kristensen, Anya Tsalenko, Jurgen Geisler, Anne Faldaas, Grethe Irene Grenaker, Ole Christian Lingjærde, Ståle Fjeldstad, Zohar Yakhini, Per Eystein Lønning, Anne-Lise Børresen-Dale

**Affiliations:** 1 Department of Genetics, Institute of Cancer Research, the Norwegian Radium Hospital, 0310 Oslo, Norway; 2 Agilent Technologies, Palo Alto, CA, USA; 3 Department of Oncology, Haukeland Hospital, Bergen, Norway; 4 Department of Informatics, University in Oslo, Norway; 5 Interagon AS, Trondheim, Norway; 6 University in Oslo, Faculty Division Radiumhospitalet, Oslo, Norway

## Abstract

**Background:**

Complex traits, which are under the influence of multiple and possibly interacting genes, have become a subject of new statistical methodological research. One of the greatest challenges facing human geneticists is the identification and characterization of susceptibility genes for common multifactorial diseases and their association to different quantitative phenotypic traits.

**Results:**

Two types of data from the same metabolic pathway were used in the analysis: categorical measurements of 18 SNPs; and quantitative measurements of plasma levels of several steroids and their precursors. Using the combinatorial partitioning method we tested various thresholds for each metabolic trait and each individual SNP locus. One SNP in *CYP19*, 3UTR, two SNPs in *CYP1B1 *(R48G and A119S) and one in *CYP1A1 *(T461N) were significantly differently distributed between the high and low level metabolic groups. The leave one out cross validation method showed that 6 SNPs in concert make 65% correct prediction of phenotype. Further we used pattern recognition, computing the p-value by Monte Carlo simulation to identify sets of SNPs and physiological characteristics such as age and weight that contribute to a given metabolic level. Since the SNPs detected by both methods reside either in the same gene (*CYP1B1*) or in 3 different genes in immediate vicinity on chromosome 15 (*CYP19, CYP11 *and *CYP1A1*) we investigated the possibility that they form *intragenic *and *intergenic *haplotypes, which may jointly account for a higher activity in the pathway. We identified such haplotypes associated with metabolic levels.

**Conclusion:**

The methods reported here may enable to study multiple low-penetrance genetic factors that together determine various quantitative phenotypic traits. Our preliminary data suggest that several genes coding for proteins involved in a common pathway, that happen to be located on common chromosomal areas and may form *intragenic *haplotypes, together account for a higher activity of the whole pathway.

## Background

The challenge of identification and characterization of susceptibility genes for complex multifactorial diseases is partly due to the limitations of parametric statistical methods for detection of gene effects that are dependent solely or partially on interactions with other genes and with environmental exposures [1,2]. These limitations are reduced by non-parametric methods such as the combinatorial partitioning method (CPM) [3], which has been used to study the effect of many marker loci on quantitative phenotypes. The focus of the method is to form subsets of loci or genotypic partitions within which the trait variability is much lower than between the partitions [3]. The loci in such a set of genotypic partitions are then selected as candidates to influence the given trait and are then cross-validated.

A modification of this method is the multifactor dimensionality reduction (MDR) method, which has been used to study the impact of multiple loci on categorical endpoints such as presence or absence of disease or response to treatment. This is accomplished by reducing the dimensionality of the multilocus data where genotypes from multiple loci are pooled into high-risk and low-risk groups, depending on whether they are more common in affected or in unaffected individuals [4,5]. This approach is so far limited to categorical parameters and cannot be applied to quantitative traits. The only possible approach to association mapping would then be to search for patterns of genotypes at different loci. Pattern recognition by machine learning techniques may then be applied to define pattern frequencies or relationships in a data set [6].

**Figure 1:**
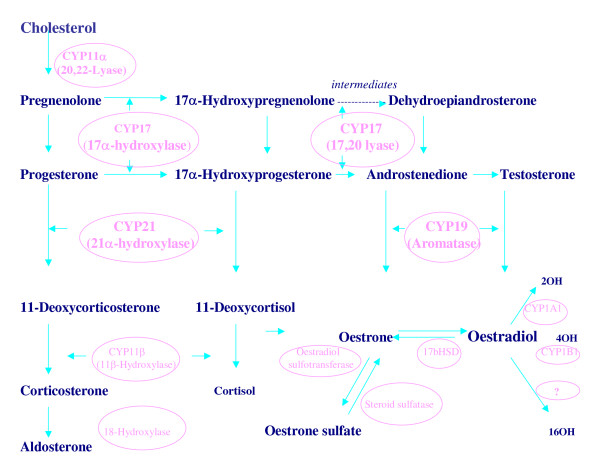
The estradiol metabolic pathway. Estradiol is synthesized from cholesterol in a series of consecutive hydroxylation relations.

In the present study we have used a variation of the combinatorial partitioning method and compared that to a pattern recognition method by the machine learning approach to identify subsets of SNPs that may predict the levels of metabolites in the estradiol metabolic pathway in healthy post-menopausal women. We have chosen this pathway since a positive correlation between estradiol exposure and risk of breast cancer among postmenopausal women has been rather well documented [7,8], and a significant correlation between plasma estrogen levels and subsequent risk of breast cancer development has been repeatedly described [9-12]. Estrone is synthesized from cholesterol in a cascade of subsequent hydroxylations [13] (Figure 1). After ovary seizure at menopause, the peripheral aromatization of androgens, mainly androstenedione into estrone, becomes the main source of circulating estrogen contributing to tumor stimulation [14]. A complex system of enzymes is responsible for estradiol synthesis and its further metabolism: *CYP17, CYP11a, CYP19, 17 β-hydroxysteroid hydrogenase, steroid sulfatase (STS), sulfotransferase (EST), CYP1A1, CYP1B1, Catechol-O-methyltransferase *([15-17] (Figure 1). Polymorphisms in these enzymes have previously been associated with both breast cancer risk and estradiol levels [2]. In the present report we have studied genetic polymorphism in all these enzymes and addressed the methodological challenge of the analysis of multiple loci 1) by free combinatorial approaches 2) in relation to intergenic haplotype structures within a common biochemical pathway.

## Results

The levels of 9 metabolites of the estradiol pathway were studied (Figure 1). High correlation was observed between the levels of the different metabolites in the plasma of healthy individuals, metabolites upstream (DHEA, DHEA-S, androstenedione and estrone) as well as downstream (estrone, estradiol, estrone- sulphate) in the pathway (Table 2). Weight and body mass index significantly correlated with the levels of estrone and estradiol, while levels of DHEA and DHEA-S inversely correlated with age. Testosterone levels correlated with height (Table 3).

### Chi square analysis

A total of 18 SNPs in 10 genes were genotyped in 109 individuals resulting in a total of 1962 genotypes. The genotype distribution of the studied polymorphisms was significantly different between the groups of individuals with metabolic activities below and above median when Chi square test was applied. The levels of E_1 _and E_2 _were significantly associated with two polymorphisms in the 3'UTR of *CYP19 *as well as two non-synonymous substitutions in the *CYP1B1 *-R48G and A119S (Table 4). The El level was also associated to the T461N SNP in *CYP1A1*. Several other non-significant trends were observed.

### Combinatorial partitioning analysis (Mutual Information Score (MIS))

**Figure 2:**
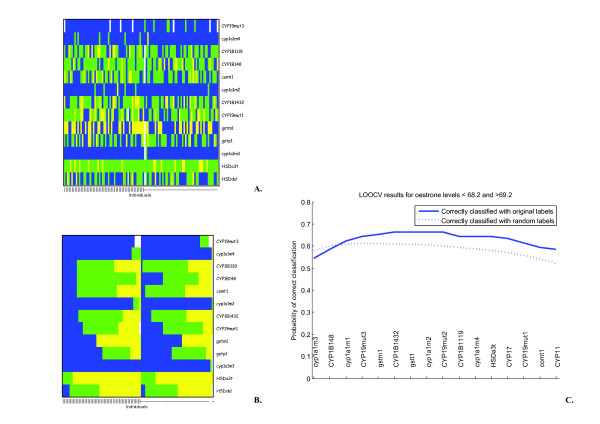
(A). Identification of minimal combination of SNPs for maximal prediction value of metabolic expression level of El (estrone) using leave one out cross validation analysis. Estrone levels <68.2 pmol/l and >69.2 pmol/l are best predicted by the genotypes in the given order (p < 0.05). Frequency distribution of the genotypes at each locus: Blue – common homozygous genotype, green – heterozygous, yellow – rare homozygous genotype for each locus (row) and each person (column), white- missing value (A). Columns under heat map labeled by '|' correspond to samples with estrone level < 68.2 pmol/l, columns labeled by '-' correspond to sample with estrone level > 69.2 pmol/l. Columns are ordered with respect to increasing estrone level (B). Stacked diagrams of frequency distribution of the genotypes at each locus (C).

The metabolic groups were further re-defined by using other thresholds than the median, using either one optimal threshold, (partitioning A) or two optimal thresholds (Partitioning B). Several moderately significant SNPs using the optimal thresholds approach were found (all SNPs with p-value < 0.05, Table 5). Leave one out cross validation analysis was performed to find sets of genotypes that jointly predict the value of the trait (high or low levels). Estrone levels partitioned into samples with values < 68.2 pmol/l and >68.2 pmol/l revealing a maximal difference in genotype distribution. A graphical representation (infogram) of the genotypes for each locus and this partition is shown in Figure 2A, where each row corresponds to a SNP and each column – to a sample. Figure 2B shows the stack diagram. The leave one out cross validation method showed that while one SNP can make only ~50% correct predictions of the estrone levels at this partition, combining 6 SNPs, including *CYP1A1m4, CYP1B1*A119S, *CYP1A1m2, CYP19utr3' *SNPS, *GSTP1, COMT *allows 65% correct prediction (Figure 2C). Two of the selected polymorphisms were known to be functional at the metabolic level from previous studies *in vitro*. In the case of random labels, the probability of finding a set of SNPs that can make better prediction was found to be 0.16 based on 100 simulations.

**Figure 3:**
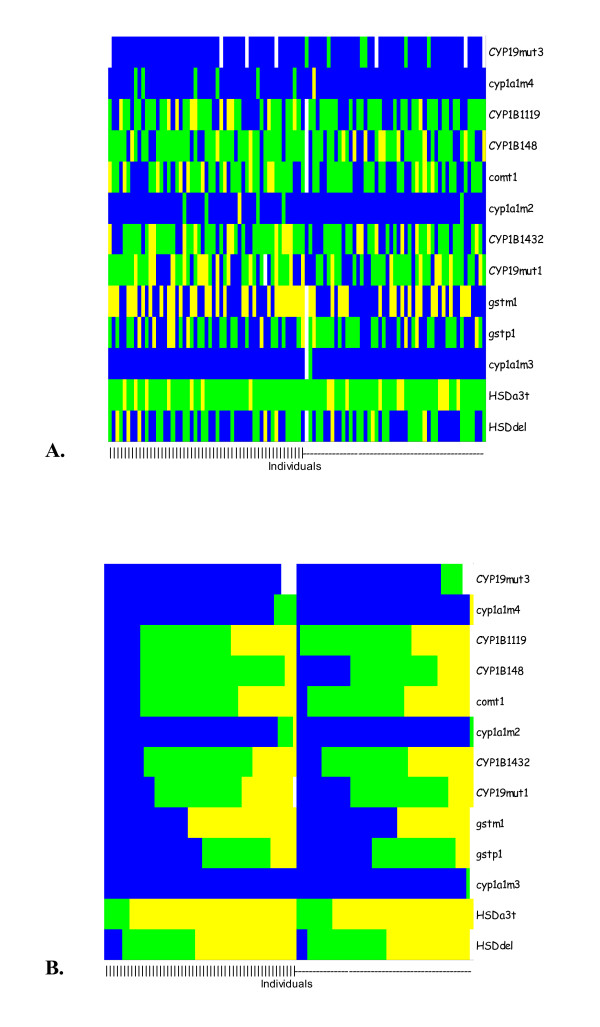
(A). Identification of minimal combination of SNPs for maximal prediction value of metabolic expression level of DHEA using leave one out cross validation analysis: women with DHEA_SO4 level>92.6 pmol/l vs women with DHEA_SO4 levels<92.6 pmol/l (p < 0.042). Frequency distribution of the genotypes at each locus: Blue – common homozygous genotype, green – heterozygous, yellow – rare homozygous genotype for each locus (row) and each person (column). Columns labeled by '|' correspond to samples with estrone level < 92.6, columns labeled by '-' correspond to sample with estrone level > 92.6 pmol/l. (B). Stacked diagrams of frequency distribution of the genotypes at each locus.

Locus *CYP11A1 *was a microsatellite repeat with 10 variant repeat length alleles. All variant alleles were categorized together: A1/A1 (wt/wt), A1/mut, mut/mut. The QT scores and p-values for this locus and each metabolite were calculated and significant differences were found for several of the metabolites. The variant allele was more frequent in women with DHEA-S level>92.6 μg/dl than in women with DHEA-S levels<92.6 μg/dl (p < 0.042). In the two threshold analysis the significance was even higher (p = 0.004) when comparing individuals with DHEA-S levels<69.7 μg/dl to those with DHEA-S levels>92.6 μg/dl (Figure 3A); stack diagram is shown in Figure 3B. For estrone the p-value was 0.008 when comparing groups of women with estrone >133 pmol/l to those with estrone< 133 pmol/l. Four women with estrone level > 133 pmol/l have genotypes A2/A2 and A4/A4. Similar to estrone, levels of estradiol were associated with the *CYP11A *variants when comparing individuals with estradiol > 33 pmol/l to those with estradiol < 33 pmol/l (p < 0.06).

### Pattern recognition of SNPs in relation to hormone metabolizing enzymes

**Figure 4:**
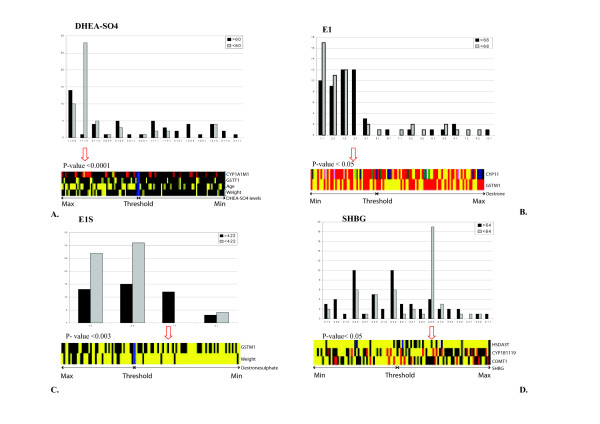
Pattern recognition: Search for combination of SNPs and physiological parameters associatedwith a given metabolic trait. On top bar diagrams illustrating the distribution of all possible combinations of traits in the metabolic groups below or a bove a given threshold (given in a sqare frame above each bar diagram). (A) **DHEA-SO4 levels ***Cyp1a1m1 *= mut/mut and *GSTT1 *= mut and Age>64 and Weight<75, *CorrMAX *0.54(49/56) p < 0.0001, Age – Weight, *CorrMAX *0.44 p < 0.002 Infogram: *CYP1A1m1: *Black = wt/wt Yellow = mut/mut Red = wt/mut; *GSTT1 *Black= wt/wt or wt/mut Yellow = mut/mut; Age Yellow = <64 Black = >64; Weight Yellow = >75 Black = <75 (B). **E1 (Estrone) levels: **The genotype combination *CYP11 *= mut and *GSTM1 *= wt is present among 12 patients with a level above 68 pmol/l, but non of the patients with a level below 68 pmol/l, *CorrMAX *0.36(53/52) p < 0.05. (C) **Estrone sulphate**, *GSTM1 *– Weight(75 kg), *CorrMAX *0.43(43/62), p < 0.003 Infogram1: -*GSTM1 *Black = wt/wt or wt/mut Yellow = mut/mut; Weight Yellow = <75 Black = >75. (D) **Sex hormone binding globulin**, *HSDA3T *= mut/mut-*CYP1B1A119S *= mut/mut-comt1 = mut/mut. *CorrMAX *0.38(59/49), p < 0.05 Infogram: *CYP1B1A119S *Red = wt/wt, Yellow = mut/mut, Black = wt/mut, *COMT1: *Red = wt/wt, Yellow = mut/mut, Black = wt/mut.

The optimal threshold of the metabolic levels was found by multiple testing close to the median. Interactions between set of SNPs and physical characteristics like age and weight was identified (Fig 4A,B). Carriers of the wt *CYP1A1m1 *and wt *GSTT1 *with age above 64 years and with a body weight above 75 kg were more often in the lower level group of the metabolite DHEA-SO4 (*CorrMAX *0.54(49/56), P-value < 0.0001) (Figure 4A). An interaction between the levels of this metabolite and age and weight (r = 0.44, p < 0.002), also seen by the conventional Chi square analysis (Table 2), was detected by this method. Individuals with weight higher than 75 kg carrying the wt *GSTM1 *had significantly higher plasma levels of E_1_S, (*CorrMAX *0.43(43/62), p < 0.003) (Figure 4B).

Another pattern of SNPs was found correlated to the estrone level; the variant allele in the 5' flanking area of *CYP11 *in combination with the wt *GSTT1 *was present among 12 individuals with a plasma level of estrone above 68 pmol/l, while none of the individuals with E_1 _plasma level below 68 pmol/l carried this combination (*CorrMAX *0.36(53/52) P-value 0.05) (Figure 4C). Individuals homozygous for the variant alleles in the *HSD17β *(A3T), *CYP1B1 *A119S, and *COMT1 *had significantly higher levels of sex hormone binding globulin, (*CorrMAX *0.38(59/49) P-value 0.05) (Figure 4D). A colored infogram illustrating the significant differences in SNP patterns above and below the different thresholds is given below each frequency diagram.

### Haplotype analysis

**Figure 5:**
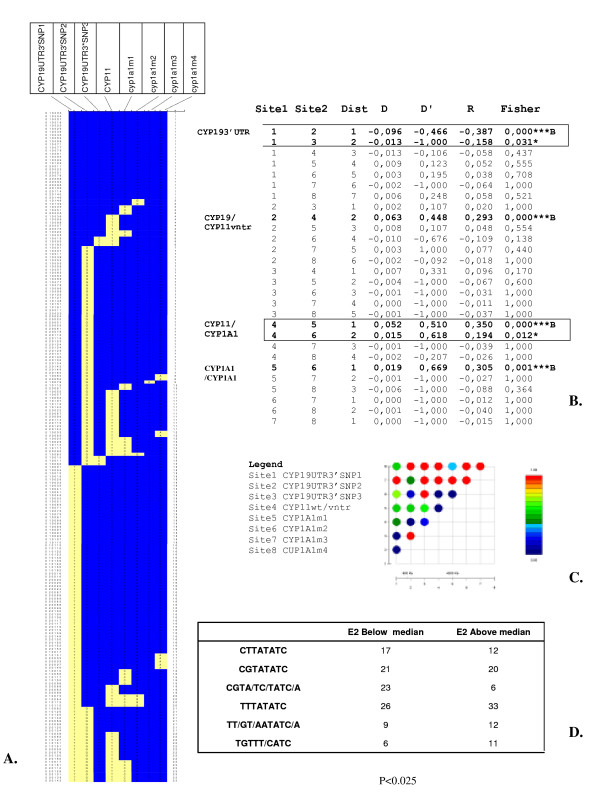
Linkage disequilibrium between SNPs in 3 genes from the estradiol metabolising pathway situated within 1 cM on chromosome 15: *CYP19*, *CYP11 *and *CYP1A*. Samples in each row, variants in columns, high frequency allele – blue, low frequency allele- yellow **(A)**. LD was observed between the 3 SNPs in the 3'UTR of *CYP19 *and 2 SNPs in *CYP1A1 *and the variant allele of *CYP11 *with one SNP in CYP19 and another in *CYP1A1*, bold in panel **(B)**. D', R and Fisher exact test values for all 8 SNPs in this chromosomal area (Site 1–8) are given in panel **(B) **and Fisher exact test results in colour diagram – blue approximating 0.00**(C)**. Individuals carrying these haplotypes had more often estradiol levels above median **(D)**.

**Figure 6:**
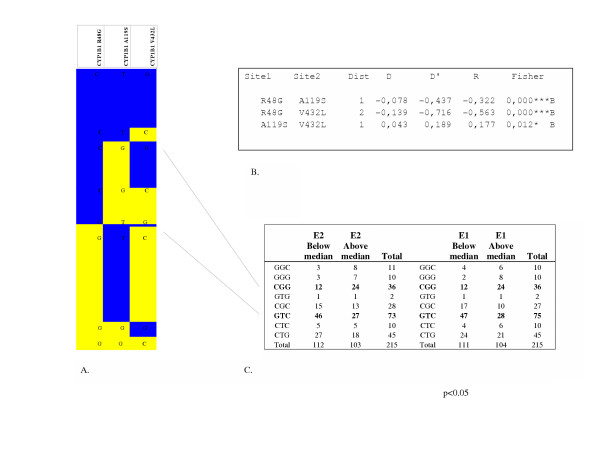
Haploptypes of 3 functional SNPs in CYP1B1, samples in each row, variants in columns, high frequency allele – blue, low frequency allele- yellow **(A)**, the extent of linkage disequilibrium, D, D', R and Fisher exact test **(B) **and association to estradiol levels **(C)**. The number of individuals representing each haplotype group with estrone (E1) and estradiol (E2) levels above and below median are given in the table **(C)**.

Since some of the SNPs detected by the above methods reside in 3 different genes in vicinity on chromosome 15 we hypothesized that they could form common *intragenic *haplotypes, which in concert might account for a higher activity of the whole pathway. Our findings suggest that the SNPs in *CYP19, CY11 *and *CYP1A1 *are not inherited at random but form common haplotypes (Figure 5A). Individuals with variant number of repeats in the microsatellite repeat of *CYP11 *were also carriers of the variant alleles in both loci *CYP1A1mi *and *m2 *(D' 0.350 and 0.194, p < 0.001 (Bonferroni corrected) and p < 0.012, respectively) as well as in *CYP19utr3' *SNP2 (D' 0.293, p < 0.001 (Bonferroni corrected) (Figure 5B). A schematic presentation of the D' values is given in Figure 5C. High D' values and significant LD was observed in addition between the 3 SNPs in *CYP19 *and the 2 of the 4 SNPs in *CYP1A1 *Carriers of the haplotype CTTATATC and CGTA(T)C(T)ATC(A) had more often E_2 _levels below median, while carriers of the TGTTT(C)ATC more often had E_2 _levels above the median (p < 0.025) (Figure 5D). The SNPs in *CYP1B1 *were also in strong linkage disequilibrium forming steady haplotype blocks (Figure 6A,B). While the haplotype CGG, containing the C allele in *CYP1B1*R48G and the G alleles in *CYP1B1*A119S and *CYP1B1 *V432L was associated with high levels of E_1 _and E_2_, the haplotype GTC containing the alternative alleles in locus was associated with lower than median levels (p < 0.05).

## Discussion

Finding effects of groups of SNPs on metabolite levels is complex since the effects of individual SNPs are small and the number of possible SNP combinations is large. We applied two different methods to help identify sets of SNPs correlated to metabolite levels: one using direct two-way classification based on combination of genotypes at selected loci, and another based on leave-one-out-cross-validation analysis. Direct classification method requires sufficiently big sample set for meaningful evaluation of genotype combination frequencies in groups with different metabolite levels. In studies like this with a small number of samples, the LOOCV method allows the evaluation of larger sets of SNPs, since the classifiers are constructed for each locus individually. In the first "pre-screening" phase of the genotype-phenotype analysis the metabolic levels were divided by median followed by sets of percentiles of the trait values. Finally, instead of pre-defining cut offs, we let the distribution of the genotypes lead us to those cut offs with a maximal difference in allele distribution. Interestingly, often these best thresholds converged to the median, i.e. for estrone in both the Mutual Information Score method as well as the pattern recognition. Whether or not these resulting cut offs have some physiological significance, remains to be investigated.

Long term exposure to estradiol increases the risk of breast cancer. The mechanisms responsible for this effect have not been firmly established. The prevailing theory proposes that estrogens increase the rate of cell proliferation by stimulating estrogen receptor-mediated transcription and thereby the number of errors occurring during DNA replication [19,20]. An alternative hypothesis proposes that estradiol can be metabolized to quinone derivatives, which can react with DNA and then remove bases from DNA through a process called depurination. Error prone DNA repair then results in point mutations [21]. These two processes, increased cell proliferation and genotoxic metabolite formation, may act in an additive or synergistic fashion to induce cancer. It has been suggested that measuring total E2 concentration and SHBG concentration may be sufficient in large epidemiological studies [12]. Our study shows that even in a small size it is sufficient to monitor only few metabolites as we observed tight correlations between them. Several genetic polymorphisms that may influence estradiol metabolism have been associated with different hormone levels. A polymorphism in *CYP19*, a 3-bp deletion in intron 4 (TTTA)_*n *= 7-3_, and a base substitution in exon 3 (G – >A) have been reported to be associated with levels of estradiol [22,23]. Genetic polymorphism in the enzymes further hydroxylating estradiol and conjugating its metabolites has also been studied. Women carrying the *COMT *Met/Met genotype had 28% higher 2-hydroxyestrone (*P = *0.08) and 31% higher 16α-hydroxyestrone concentrations (*P= *0.02), compared to women with the Val/Val genotype [22].

The previous studies discussed above analyzed single loci. In a recent breast cancer case -control study, including categorical values only (genotypes), a four-locus susceptibility model including the polymorphisms of *COMT, CYP1A1m1, CYP1B1 *codon 48, and *CYP1B1 *codon 432 was found associated with breast cancer [2]. The four-locus model was significant at the p = 0.001 level by permutation testing bringing evidence of epistasis, or gene-gene interaction in the case-control setting. Each genotype at a particular locus had an influence on breast cancer disease risk dependent on the genotypes at each of the other three loci. With that in mind, we searched the hormone metabolic pathway for interactions between these loci both at random, combinatorial, without regards to the chromosomal localisation and with regards to the LD in genes that may form common haplotype structures. Two such domains were identified – the polymorphisms in *CYP1B1 *form stable haplotype structures in Norwegian population (Zimarina et al, submitted), and a block on the long arm of chromosome 15 consisting of *CYP11A1 *gene close to the *CYP1A1 *(less than 1 cM) gene and approximately 27.4 cM telomeric to the *CYP19 *gene. Indeed, we found 2 haplotypes in the *CYP1B1*, which were significantly overrepresented in individuals with El and E2 levels above median. Two of the three genotypes (R48G and V432L) were among the best predictors according to the combinatorial partitioning method as well. Furthermore, the same *CYP1B1 *haplotypes (CGG and GTC) were associated with breast cancer risk (Zimarina et al, submitted). The CGG haplotype includes both the V432 form of *CYP1B1 *and the R48 with higher 4-OH/2-OH E2 metabolic ratio and affinity (Km) towards 17b-estradiol respectively [24,25]. More unexpectedly, we found high D' values among SNPs residing in different genes but coding for proteins in the same metabolic pathway. The haplotype comprising of the T allele of *CYP19utr3' *SNP1, the variant number of repeats of *CYP11 *and the variant alleles of *CYP1A1m1 *and *m2 *we associated with high E2 levels (above median). Upregulation of *CYP1A1 *by dioxin derivatives through the Ah receptor leads to down-regulation of *CYP19 *and *ER*. The close location of these genes gives an attractive opportunity to study whether they are regulated by a common regulatory unit. The haplotype structures may vary from population to population – hence explain the variability of the published data on various susceptibility alleles in a number of genes.

The fact that we manage to predict correctly 65% of the individuals according to their metabolic levels based on this limited selection of SNPs in healthy individuals, make us believe that we have identified markers of estradiol levels in the present study. Furthermore, we found similar combinations of SNPs as those involved in the susceptibility combinations from the case control study [2]. We are presently conducting a larger study of both cases and controls with a higher SNP density to improve our 65% prediction value. In the last stage of the preparation of this manuscript another large study of 1975 individuals was published [26] confirming our previous initial report of an association of the polymorphism in *CYP193UTR *and aromatase activity [27] and in concordance with our present observation of association with plasma levels. Here we demonstrate that the association can be discovered in much smaller number of individuals (109) using the multilocus data analysis.

## Conclusion

These studies provide further evidence that genetic variation may appreciably alter plasma level of sex hormone and thus have an effect on disease risk. We describe an approach for multilocus approach to study multiple low-penetrance genetic factors that together determine quantitative phenotypic traits.

## Methods

Blood samples were collected from 109 healthy female volunteers between 55 and 75 years of age on a regular mammographic screening. The women enrolled had 2 consecutive negative mammograms in a period of 2 years and were not on hormone replacement therapy (HRT). The plasma levels of the metabolites E_1_, E_2_, E_1_S, DHEA, androstenedione and testosterone were analysed as described previously in [28]. DNA was isolated from EDTA blood using chloroform/phenol extraction followed by ethanol precipitation according to standard procedures using the Applied Biosystems 340A Nucleic Acid Extractor.

### Genotyping

Primer sets and methods for analysis are summarized in Table 1 and as described in [29,30].

### Statistical Analysis

#### Parametric method

Metabolic levels for each metabolite were divided into below and above median and allele and genotype frequency distributions were compared using the Chi square test.

#### Non-parametric methods

##### Combinatorial partitioning method

To relax the choice of a metabolite level percentile at which to partition the set of samples, we seek an optimal threshold for each metabolite and each SNP. This optimality is defined by an information theoretic measure of concordance between the partition of the sample set according to genotypes and the partition of the sample set defined by the two sides of a metabolite level threshold. The methods we applied in this dataset are an adaptation of the combinatorial approach of [3] and are briefly described below.

Let (*x*_*i*_*, q*_*i*_), *i *= l,2,...,*n *denote the measurements for a particular pair consisting of a SNP and a quantitative variable (metabolite level) across all patients. For any such pair we seek a threshold *t*, such that the genotype frequencies in the samples with *q<t *will be the most different from the genotype frequencies in the samples with *q≥t*. This difference is measured by the mutual information score. The search process itself is exhaustive, considering all possible thresholds. More precisely, for a SNP locus *L *and a quantitative trait *q*, let G be a partition of the sample-set induced by the genotypes at locus *L*. For a threshold *t*, let *C*_*t *_be a binary partition of the sample-set defined by *q<t *and *q≥t*. The Mutual Information Score (*MIS*) is defined as the difference between the entropy of the partition *C*_*t *_and the entropy of *C*_*t *_conditioned on *G*:

*MIS(C*_*t*_*, G) = H(C*_*t*_*) - H(C*_*t*_/*G*),

where *H *is the entropy or conditional entropy function, as appropriate. The best threshold t^
 MathType@MTEF@5@5@+=feaafiart1ev1aaatCvAUfKttLearuWrP9MDH5MBPbIqV92AaeXatLxBI9gBaebbnrfifHhDYfgasaacH8akY=wiFfYdH8Gipec8Eeeu0xXdbba9frFj0=OqFfea0dXdd9vqai=hGuQ8kuc9pgc9s8qqaq=dirpe0xb9q8qiLsFr0=vr0=vr0dc8meaabaqaciaacaGaaeqabaqabeGadaaakeaacuWG0baDgaqcaaaa@2E2D@ for a pair (L, *q*) is such that

MIS(Ct^,G)
 MathType@MTEF@5@5@+=feaafiart1ev1aaatCvAUfKttLearuWrP9MDH5MBPbIqV92AaeXatLxBI9gBaebbnrfifHhDYfgasaacH8akY=wiFfYdH8Gipec8Eeeu0xXdbba9frFj0=OqFfea0dXdd9vqai=hGuQ8kuc9pgc9s8qqaq=dirpe0xb9q8qiLsFr0=vr0=vr0dc8meaabaqaciaacaGaaeqabaqabeGadaaakeaacqWGnbqtcqWGjbqscqWGtbWucqGGOaakcqWGdbWqdaWgaaWcbaGafmiDaqNbaKaaaeqaaOGaeiilaWIaem4raCKaeiykaKcaaa@3688@ = *max*_*min*(*q*) ≤*t*≤*max(q)*_*MIS(C*_*t*_*, G).*

The corresponding *p*-values were also calculated, effectively counting all possible ways of partitioning the samples that would give a score better than MIS(Ct^,G)
 MathType@MTEF@5@5@+=feaafiart1ev1aaatCvAUfKttLearuWrP9MDH5MBPbIqV92AaeXatLxBI9gBaebbnrfifHhDYfgasaacH8akY=wiFfYdH8Gipec8Eeeu0xXdbba9frFj0=OqFfea0dXdd9vqai=hGuQ8kuc9pgc9s8qqaq=dirpe0xb9q8qiLsFr0=vr0=vr0dc8meaabaqaciaacaGaaeqabaqabeGadaaakeaacqWGnbqtcqWGjbqscqWGtbWucqGGOaakcqWGdbWqdaWgaaWcbaGafmiDaqNbaKaaaeqaaOGaeiilaWIaem4raCKaeiykaKcaaa@3688@[3].

In a parallel approach to assessing SNP-metabolite association we tried all possible pairs of thresholds from the set of 5th, 10th, 15th, 20th, 25th etc percentiles of the trait values. For a pair of thresholds a and b, we considered the partition *C*_*a*,*b *_of the samples into samples with trait *q ≤ a*, samples with *a<q<b *and samples with *q≥b*. For each such partition, we computed the corresponding mutual information scores *MIS(C*_*a,b*_, *G*) and picked the pair of thresholds (a^,b^
 MathType@MTEF@5@5@+=feaafiart1ev1aaatCvAUfKttLearuWrP9MDH5MBPbIqV92AaeXatLxBI9gBaebbnrfifHhDYfgasaacH8akY=wiFfYdH8Gipec8Eeeu0xXdbba9frFj0=OqFfea0dXdd9vqai=hGuQ8kuc9pgc9s8qqaq=dirpe0xb9q8qiLsFr0=vr0=vr0dc8meaabaqaciaacaGaaeqabaqabeGadaaakeaacuWGHbqygaqcaiabcYcaSiqbdkgaIzaajaaaaa@3044@) that gave maximum score. The calculated p-values take into account the multiple search over all possible thresholds as described in [3].

Further, for each SNP-trait pair, and a partition of the sample-set *C *defined by a single best threshold or by a pair of best thresholds, we ran leave one out cross validation analysis in order to find a set of SNPs such that genotypes at these SNPs can jointly predict the classification of samples with respect to *C*, i.e. whether the value of the quantitative trait level is above or below threshold [3]. Leave one out cross validation analysis, working with a given subset of SNPs, *S*, consists of the following steps:

1. Hide a sample

2. For each SNP in the subset *S*, construct a classifier based on the likelihood of each class of the partition *C *given the genotypes of remaining (non hidden) samples at this locus

3. Classify the hidden sample using the sum of single-SNP classifiers

4. Repeat steps 1–3 for each sample, and thus determine the number of correct predictions for the subset *S*

Eventually, we also seek a subset *S *with the best performance. Several search techniques were used for finding best subset of SNPs in order to avoid evaluating each possible subset for each locus/trait pair. These techniques included ordering SNPs by mutual information score and evaluating sets of top scoring SNPs, as well as performing forward and backward sequential searches [3]. Further, we estimated the probability of finding such predictive subsets of SNPs for random labels by simulations.

##### Pattern recognition by the two way classification method

The problem of finding an association between groups of individuals with metabolic levels above or below a certain threshold, referred to as the positive and negative groups, and combinations of genotypes at specific polymorphic loci may be formulated as a two-way classification problem. When evaluating a specific combination of genotypes against a particular patient record, the outcome can be a true-positive (i.e., the individual has all the genotypes in the given combination, and the patient is in the positive group), a true-negative (i.e., the patient does not have the specific combination of genotypes, and the patient is in the negative group), a false-positive (i.e., the individual has all the genotypes the given combination, and the patient is in the negative group) and a false-negative (i.e. the patient does not have the specific combination of genotypes and the patient is in the positive group). For a two-way classification problem, the correlation of a specific combination of genotypes for each combination of SNPs may be computed as

C=NtpNtn−Nfn+Nfp(Ntn+Nfn)(Ntn+Nfp)(Ntp+Nfn)(Ntp+Nfp)
 MathType@MTEF@5@5@+=feaafiart1ev1aaatCvAUfKttLearuWrP9MDH5MBPbIqV92AaeXatLxBI9gBaebbnrfifHhDYfgasaacH8akY=wiFfYdH8Gipec8Eeeu0xXdbba9frFj0=OqFfea0dXdd9vqai=hGuQ8kuc9pgc9s8qqaq=dirpe0xb9q8qiLsFr0=vr0=vr0dc8meaabaqaciaacaGaaeqabaqabeGadaaakeaacqWGdbWqcqGH9aqpdaWcaaqaaiabd6eaonaaBaaaleaacqWG0baDcqWGWbaCaeqaaOGaemOta40aaSbaaSqaaiabdsha0jabd6gaUbqabaGccqGHsislcqWGobGtdaWgaaWcbaGaemOzayMaemOBa4gabeaakiabgUcaRiabd6eaonaaBaaaleaacqWGMbGzcqWGWbaCaeqaaaGcbaWaaOaaaeaadaqadaqaaiabd6eaonaaBaaaleaacqWG0baDcqWGUbGBaeqaaOGaey4kaSIaemOta40aaSbaaSqaaiabdAgaMjabd6gaUbqabaaakiaawIcacaGLPaaadaqadaqaaiabd6eaonaaBaaaleaacqWG0baDcqWGUbGBaeqaaOGaey4kaSIaemOta40aaSbaaSqaaiabdAgaMjabdchaWbqabaaakiaawIcacaGLPaaadaqadaqaaiabd6eaonaaBaaaleaacqWG0baDcqWGWbaCaeqaaOGaey4kaSIaemOta40aaSbaaSqaaiabdAgaMjabd6gaUbqabaaakiaawIcacaGLPaaadaqadaqaaiabd6eaonaaBaaaleaacqWG0baDcqWGWbaCaeqaaOGaey4kaSIaemOta40aaSbaaSqaaiabdAgaMjabdchaWbqabaaakiaawIcacaGLPaaaaSqabaaaaaaa@6C23@

Where *Ntp, Ntn, Nfn*, and *Nfp*, denotes the number of patient records which are respectively true-positive, true-negative, false-negative, and false-positive. The significance of a specific combination of polymorphisms with correlation *CorrMAX *is defined as the probability of finding a better, or just as good, combination of polymorphisms by random. This probability is referred to as the p-value, and is computed by Monte Carlo simulation. The steps for the Monte Carlo procedure are as described in [31]. Briefly, for each individual record, permute the "above" or "below" labels randomly from the same distribution as in the original data. Calculate *CorrMAX *for the permuted data. If *CorrMAX *for the permuted data is larger than, or equal to *CorrMAX *from the original data, count 1; otherwise count 0. Repeat steps 1, 2 and 3 *k *times. Estimate the p-value, the total count for *CorrMAX *divided by the total number of shuffles *k*. When computing *CorrMAX *for the permuted data, we include all polymorphisms, not only the polymorphisms in the combination to test the significance of. The standard value for *k*(number of repeated steps) is 2000.

#### Haplotype analysis

Haplotypes were estimated using PHASE2.0 software. Linkage disequilibrium and D and D' values were calculated using DNAsp software. Significance of the linkage disequilibrium (LD) was estimated using Fisher exact test with Bonferoni correction for the final p-value.

## Aurhor's contribution

VNK, final design of the study, supervised AF, carried out the haplotype analysis and drafted the manuscript

AT, carried out the Mutual Information Score and LOOCV analyses

JG, carried out the metabolic profiling

AF, performed the genotyping as a part of her master's project

GIGA, provided technical assistance in the lab and isolated the DNA

OCL, consulted the statistical analysis and comparison of the methods

SF, performed the pattern recognition analysis

ZY, supervised the statistical analysis and edited the manuscript

PEL collected the blood samples and participated in designing the study

ALBD designed the study and DNA preparation

All authors have read and edited the manuscript and approve the final manuscript.

## Figures and Tables

**Table 1:** Summary of the selected SNPs and the respective method of genotyping. Gene/SNP, rs number given when available, aminoacid change or UTR. Primer set: for the assyas developed for this study or otherwise as referred to original publication. Method of analysis: platform and assay selection.

**Gene/SNP**	**Primer set**	**Method of analysis**
*CYP11A1, repeat*	f(5'-6-FAM-GTC-AGC-TGT-ACT-GAA-TTA-CAG-CC-3') r(5'-TCT-TGC-CAG-CTT-GGG-CAA-CAT-ACG-3'	Fragment analysis ABI310, (Applied Biosystems)
*CYP17, rs 743572*	f 5'-CAT-TCG-CAC-CTC-TGG-AGT-3' r 5'-GGC-TCT-TGG-GGT-ACT-TG-3'	RLFP *Msp*A1 (Promega),
*CYP19 3'UTRSNP1, rs10046*	5'-ATA TTC TGG CAA CTG TCT-3' 5'-GAG GAT GAC ACT ATT GGC-3'	BDT (Big Dye Terminator) sequencing ABI310 (Applied Biosystems)
*CYP19 3'UTRSNP2, rs4646*	5'-GAC AGT GTG TTG AGA GCA TAC AGA-3' 5'-TTC TTC ACC GAC TAT TTC TC-3'	BDT (Big Dye Terminator) sequencing ABI310 (Applied Biosystems)
*CYP19 3'UTRSNP3, rs2255192*	5'-CAT TGA ATC ATT GTA TGT GGT CAT-3' 5'-GGA AAT GGG ATG GAA ATA GAT TAC A-3'	BDT (Big Dye Terminator) sequencing ABI310 (Applied Biosystems)
*HSD-A3T, rs3138620*	5'-6-FAM CAG TAC TAA AGG CCC TAT TAT CAA A-3' 5'-AGG CTG CAG TGA GTC CAG AT-3'	Fragment analysis ABI310, (Applied Biosystems)
*HSD-DEL, rs8191194*	5'-6-FAM GTG ACC CAC GAA ACA CAG G-3' 5'-CAG AAG GTG AAG AAC TCA TCC A-3'	Fragment analysis on a ABI310, (Applied Biosystems)
*CYP1B1 R48G*	5'-GCACCCCTGAGTGTCACGCC-3', 5'-TTCCAGTGCTCCGAGTAGTG-3'	RFLP, *Rsr II*. (Fermentas)
*CYP1B1 A119S*	5'-GCACCCCTGAGTGTCACGCC-3', 5'-TTCCAGTGCTCCGAGTAGTG-3'	RLFP, *NgoM IV *(New England BioLabs)
*CYP1B1 V432L, rs1056836*	5'-CCAGCCCAACCTGCCCTATG-3' 5'-CCAGGATGGAGATGAAGA-3'	RLFP, *Eco577 *(Fermentas)
*CYP1A1m1 T3801C*	Cascorbi et [19].	See reference
*CYP1A1m2 I462V, rs1048943*	Cascorbi et [19].	See reference
*CYP1A1mS T3205C*	Cascorbi et [19].	See reference
*CYP1A1m4 T461N, rs1799814*	Cascorbi et [19].	See reference
*COMT, rs4680*	Matsui et al. 2000, [20].	See reference
*GSTM1*	Kristensen et al 1998 [31].	See reference
*GSTT1*	Kristensen et al 1998[31].	See reference
*GSTP1*	Kristensen et al 1998[31].	See reference

**Table 2:** Correlation between metabolic levels of estradiol and its precursors in the plasma of healthy post-menopausal women. DHEA, dihydroepiandrostenedione, DHEA-SO4, dihydroepiandrostenedione sulfate, androstenedione, testosterone, E1, estrone, E2, estradiol, E1S, estrone- sulphate.

Metabolite	DHEA	DHEA-SO4	Androstenedione	Testosterone	E1	E2	E1S
DHEA	1						
DNEA-SO4	0.578 p < 0.001	1					
Androstenedione	0.494 p < 0.001	0.606 p < 0.0001	l				
Testosterone	NS	NS	0.321 p < 0.001	1			
E1	0.201 p < 0.034	0.285 p < 0.002	0.217 p < 0.021	NS	1		
E2	NS	NS	NS	NS	0.873 p < 0.0001	1	
E1S	NS	NS	NS	NS	0.776 p < 0.0001	0.795 p < 0.0001	1

**Table 3:** Association of estrogen and its precursors with age, height, weight and body mass index.

Metabolite	Age	Height	Weight	Body-mass index
DHEA	-0.199 p < 0.036	NS	NS	NS
DNEA-SO4	-0.218 p < 0.021	-0.210 p < 0.026	NS	NS
Androstenedione	NS	NS	NS	NS
Testosterone	NS	0.300 p < 0.001	NS	NS
E1	NS	NS	0.258 p < 0.006	0.191 p < 0.042
E2	NS	NS	0.349 p < 0.000	0.308 p < 0.001
E1S	NS	NS	NS	NS

**Table 4:** Statistical significance of distributions of genotypes in the listed estradiol metabolizing enzymes in below and above median levels of metabolites DHEA, dihydroepiandrostenedione, DHEA-SO4, dihydroepiandrostenedione sulfate, androstenedione, testosterone, E1, estrone, E2, estradiol, E1S, estrone- sulphate, SHBG, sex hormone binding globulin. NS – not significant.

		**Below and above median values**							
**Gene/SNP**		**DHEA**	**DHEA-SO4**	**Androstenedione**	**Testosterone**	**E1**	**E2**	**E1S**	**SHBG**
***HSD DEL***	5'flanking 12 bp deletion	NS	NS	NS	NS	NS	NS	NS	NS
***HSD A3T***	5'flanking (AAAT)n	NS	NS	NS	p < 0.053	NS	NS	NS	NS
***CYP17***	5'flanking	NS	NS	NS	NS	NS	NS	NS	NS
***CYP19UTRSNP1***	3'untranslated	NS	NS	NS	NS	p < 0.037	p < 0.048	NS	NS
***CYP19UTRSNP2***	3'untranslated	NS	NS	NS	NS	NS	NS	NS	NS
***CYP19 UTRSNP3***	3'untranslated	NS	NS	NS	p < 0.089	p < 0.014	p < 0.013	NS	NS
***CYP11***	5'flanking	NS	P = 0.042	p < 0.053	NS	p < 0.003*	p < 0.004*	NS	NS
***CYP1B1 **R48G*	coding nonsynon R48G	NS	p < 0.082	NS	NS	p < 0.049	p < 0.082	p < 0.042	NS
***CYP1B1 **A119S*	coding nonsynon A119S	NS	NS	NS	NS	p < 0.073	p < 0.002	NS	NS
***CYP1B1 **V432L*	coding nonsynon V432L	NS	NS	NS	NS	NS	NS	NS	NS
***CYP1A1 m1***	3'untranslated	NS	NS	NS	NS	NS	NS	NS	NS
***CYP1A1 m2***	coding nonsynon I462V	NS	p < 0.044	NS	NS	NS	NS	NS	NS
***CYP1A1 m3***	3'untranslated	NS	NS	NS	NS	NS	NS	NS	NS
***CYP1A1 m4***	coding nonsynon T461N	NS	NS	NS	NS	p < 0.017	NS	NS	NS
***COMT***	coding nonsynon V158M	NS	NS	p < 0.02	NS	NS	NS	NS	NS
***GSTM1***	gene deletion	NS	NS	NS	NS	NS	NS	NS	NS
***GSTT1***	gene deletion	NS	NS	NS	NS	NS	NS	NS	NS
***GSTp1***	coding nonsynon	NS	NS	NS	NS	NS	NS	NS	NS

*ANOVA analysis

**Table 5:** Searching for optimal thresholds approach to identify genotypes associated with quantitative trait (all SNPs with p-value < 0.05). Partitioning A shows significant results for one optimal threshold (two partitions), and Partitioning B shows the results for two optimal thresholds (three partitions). The calculated p-values take into account the multiple search over all possible thresholds as described in [3].

		**Partitioning A**	**Partitioning B**
**Metabolite**	**SNP**	**p-value**	**p-value**

DHEA	*CYP1B1 *R48G	NS	0.022
E1	*CYP1B1 *A119S	0.039	0.0045
E1S	*CYP1B1 *A119S	NS	0.043
Androstenedione	*CYP19 *UTRSNP1	0.042	NS
DHEA-SO4	*cyp1A1 *m1	NS	0.010
DHEA-SO4	*cyp1A1 *m2	0.022	0.011
E1	*cyp1A1 *m4	0.042	0.028

## References

[B1] Hoh J, Ott J (2003). Mathematical Multilocus Approaches to Localize Complex Human Trait Genes. Nature Reviews.

[B2] Ritchie MD, Hahn LW, Roodi N, Bailey LR, Dupont WD, Parl FF, Moore JH (2001). Multifactor-dimensionality reduction reveals high-order interactions among estrogen-metabolism genes in sporadic breast cancer. Am J Hum Genet.

[B3] Tsalenko A, Ben-Dor A, Cox N, Jakhini Z (2003). Methods for Analysis and Visualization of SNP genotype data for Complex Diseases. Pac Symp Biocomput.

[B4] Hahn LW, Ritchie MD, Moore JH (2003). Multifactor dimensionality reduction software for detecting gene-gene and gene-environment interactions. Bioinformatics.

[B5] Byng MC, Whittaker JC, Cuthbert AP, Mathew CG, Lewis CM (2003). SNP subset selection for genetic association studies. Ann Hum Genet.

[B6] Koza JR, Andre D (1996). A case study where biology inspired a solution to a computer science problem. Pac Symp Biocomput.

[B7] Nandi S, Guzman RC, Yang J (2001). Hormones and mammary carcinogenesis in mice, rats, and humans: a unifying hypothesis. Proc Natl Acad Sci USA.

[B8] Clemons M, Goss P (2001). Estrogen and the risk of breast cancer. N Engl J Med.

[B9] Endogenous Hormones and Breast Cancer Collaborative Group (2002). Endogenous sex hormones and breast cancer in postmenopausal women: Reanalysis of nine prospective studies. Journal of the National Cancer Institute.

[B10] Endogenous Hormones and Breast Cancer Collaborative Group (2003). Free Estradiol and Breast Cancer Risk in Postmenopausal Women: Comparison of Measured and Calculated Values. Cancer Epidemiol Biomarkers Prev.

[B11] Trichopoulos D, MacMahan B, Cole P (1972). Menopause and breast cancer risk. Journal of the National Cancer Institute.

[B12] Liehr JG (2001). Genotoxicity of the steroidal estrogens estrone and estradiol: possible mechanism of uterine and mammary cancer development. Apmis.

[B13] Riza E, Dos SS, De Stavola B, Bradlow HL, Sepkovic DW, Linos D, Linos A (2001). Urinary estrogen metabolites and mammographic parenchymal patterns in postmenopausal women. Cancer Epidemiol Biomarkers Prev.

[B14] Jacobs S, Lonning PE, Haynes B, Griggs L, Dowsett M (1991). Measurement of aromatisation by a urine technique suitable for the evaluation of aromatase inhibitors in vivo. J Enzyme Inhib.

[B15] Huang CS, Chern HD, Chang KJ, Cheng CW, Hsu SM, Shen CY (1999). Breast cancer risk associated with genotype polymorphism of the estrogen-metabolizing genes CYP17, CYP1A1, and COMT: a multigenic study on cancer susceptibility. Cancer Research.

[B16] Kristensen VN, Kure EH, Erikstein B, Harada N, Borresen-Dale A (2001). Genetic susceptibility and environmental estrogen-like compounds. Mutation Research.

[B17] Kristensen VN, Borresen-Dale AL (2001). Molecular epidemiology of breast cancer: genetic variation in steroid hormone metabolism. Mutation Research.

[B18] Lonning PE, Helle SI, Johannessen DC, Adlercreutz H, Lien EA, Tally M, Ekse D, Fotsis T, Anker GB, Hall K (1995). Relations between sex hormones, sex hormone binding globulin, insulin-like growth factor-I and insulin-like growth factor binding protein-1 in post-menopausal breast cancer patients. Clin Endocrinol.

[B19] Henderson BE, Feigelson HS (2000). Hormonal carcinogenesis. Carcinogenesis.

[B20] Yue W, Santen RJ, Wang JP, Li Y, Verderame MF, Bocchinfuso WP, Korach KS, Devanesan P, Todorovic R, Rogan EG, Cavalieri EL (2003). Genotoxic metabolites of estradiol in breast: potential mechanism of estradiol induced carcinogenesis. J Steroid Biochem Mol Biol.

[B21] Shelley S, Tworoger J, Chubak EJ, Aiello CM, Ulrich C, Atkinson Potter JD, Yasui Y, Stapleton PL, Lampe JW, Farin FM, Stanczyk FZ, McTiernan A (2004). Association of CYP17, CYP19, CYP1B1, and COMT Polymorphisms with Serum and Urinary Sex Hormone Concentrations in Postmenopausal Women. Cancer Epidemiol Biomarkers Prev.

[B22] Somner J, McLellan S, Cheung J, Mak YT, Frost ML, Knapp KM, Wierzbicki AS, Wheeler M, Fogelman I, Ralston SH, Hampson GN (2004). Polymorphisms in the P450 c17 (17-hydroxylase/17,20-Lyase) and P450 c19 (aromatase) genes: association with serum sex steroid concentrations and bone mineral density in postmenopausal women. J Clin Endocrinol Metab.

[B23] Shimada T, Watanabe J, Kawajiri K, Sutter TR, Guengerich FP, Gillam EMJ, Inoue K (1999). Catalytic properties of polymorphic human cytochrome P450 1B1 variants. Carcinogenesis.

[B24] Shimada T, Watanabe J, Inoue K, Guengerich FP, Gillam EM (2001). Specificity of 17 beta – estradiol and benzo[a]pyrene oxidation by polymorphic human cytochrome P450 1B1 variants substituted at residues 48, 119 and 432. Xenobiotica.

[B25] Dunning AM, Dowsett M, Healey CS, Tee L, Luben RN, Folkerd E, Novik KL, Kelemen L, Ogata S, Pharoah PD, Easton DF, Day NE, Ponder BA (2004). Polymorphisms associated with circulating sex hormone levels in postmenopausal women. J Natl Cancer Inst.

[B26] Kristensen VN, Harada N, Yoshimura N, Haraldsen E, Lonning PE, Erikstein B, Karesen R, Kristensen T, Borresen-Dale AL (2000). Genetic variants of CYP19 (aromatase) and breast cancer risk. Oncogene.

[B27] Nedelcheva Kristensen V, Andersen TI, Erikstein B, Geitvik G, Skovlund E, Nesland JM, Borresen-Dale AL (1998). Single tube multiplex polymerase chain reaction genotype analysis of GSTM1, GSTT1 and GSTP1: relation of genotypes to TP53 tumor status and clinicopathological variables in breast cancer patients. Pharmacogenetics.

[B28] Geisler J, Berntsen H, Lonning PE (2000). A novel HPLC-RIA method for the simultaneous detection of estrone, estradiol and estrone sulphate levels in breast cancer tissue. J Steroid Biochem Mol Biol.

[B29] Cascorbi I, Brockmoller J, Roots I (1996). A C4887A polymorphism in exon 7 of human CYP1A1: population frequency, mutation linkages, and impact on lung cancer susceptibility. Cancer Research.

[B30] Matsui A, Ikeda T, Enomoto K, Nakashima H, Omae K, Watanabe M, Hibi T, Kitajima M (2000). Progression of human breast cancers to the metastatic state in linked to genotypes of catechol-o-methyltransferase. Cancer Letters.

[B31] McIntyre LM, Martin ER, Simonsen KL, Kaplan NL (2003). Circumventing Multiple Testing: A Multilocus Monte Carlo Approach to Testing for Association. Genetic Epidemiology.

